# Interpreting protein dose trials in critical illness: a guide for the bedside clinician

**DOI:** 10.1186/s13054-026-06116-4

**Published:** 2026-06-27

**Authors:** Lee-anne Chapple, Julia Bels, Zheng-Yii Lee, Matthew Summers, Suzie Ferrie, Christian Stoppe, Dieter Mesotten, Adam Deane, Marcel C. G. van de Poll, Emma Ridley

**Affiliations:** 1 School of Medicine, College of Health, Adelaide University, Adelaide, South Australia Australia; 2 Intensive Care Unit, Royal Adelaide Hospital, North Terrace, Adelaide, South Australia Australia; 3 Department of Intensive Care Medicine, Maastricht University Medical Centre, Maastricht, The Netherlands; 4 School for Nutrition and Translational Research in Metabolism, Maastricht University, Maastricht, The Netherlands; 5 Department of Anaesthesiology, Faculty of Medicine, University of Malaya, Kuala Lumpur, Malaysia; 6 Department of Anaesthesiology, Intensive Care, Emergency and Pain Medicine, University Hospital Würzburg, Würzburg, Germany; 7 Department of Cardiac Anaesthesiology and Intensive Care Medicine, Charité, Berlin, Germany; 8 Department of Nutrition & Dietetics, Royal Prince Alfred Hospital, Camperdown, New South Wales Australia; 9 Faculty of Medicine and Health, University of Sydney, Sydney, Australia; 10 Department of Intensive Care Medicine, Ziekenhuis Oost-Limburg Genk, Genk, Belgium; 11 Faculty of Medicine and Life Sciences, UHasselt, LCRC, Diepenbeek, Belgium; 12 Department of Critical Care, The University of Melbourne, Melbourne, Australia; 13 Intensive Care Unit, Royal Melbourne Hospital, Parkville, Victoria Australia; 14 Department of Surgery, Maastricht University Medical Centre, Maastricht, The Netherlands; 15 Australian and New Zealand Intensive Care Research Centre, Monash University, Melbourne, Victoria Australia; 16 Dietetics and Nutrition, Alfred Hospital, Melbourne, Victoria Australia

**Keywords:** Protein, Intensive care, Nutrition, Enteral nutrition, Critical care

## Abstract

**Background:**

Critical illness induces a catabolic state, associated with profound muscle wasting and functional disability in survivors. Based on evidence at the time of development, international critical care nutrition guidelines recommend providing higher protein doses than in healthy populations.

**Main body:**

Three recent international multi-centre randomised trials (total *n* = 5633 patients) have compared higher protein doses to usual protein doses in critically ill patients. Based on the primary outcomes, these trials concluded that higher protein doses did not improve time-to-discharge alive or number of days free of the index hospital and alive at day 90, with worse functional recovery using the EQ-5D-5L health utility score over 180 days. Supported by evidence from recent trials, it appears preferable to commence protein delivery at low doses once patients are haemodynamically stable and increase progressively over the first 5 days to deliver a maximum of 1.2 g/kg/day. Based on current trial data, this upper limit may represent a safer alternative to doses >1.2 g/kg/day, acknowledging that the optimal dose may be lower. Subgroup analyses suggest that patients with an acute kidney injury may be particularly vulnerable to higher protein. There are no data to indicate the minimum protein dose that can be safely delivered to critically ill patients over their entire ICU stay. While existing trials included patients with a prolonged ICU stay, no trial identifying this cohort pre-randomisation has been conducted. It cannot be excluded that higher protein doses may provide benefit later in recovery, when the anabolic resistance to dietary protein observed early in the ICU admission has subsided. Evidence is also lacking on optimal protein targets for patients after ICU discharge.

**Conclusion:**

In critically ill adults, we suggest that protein doses be commenced at a low dose and increased progressively to a maximum of 1.2 g/kg/day based on recent randomised trials.

## Background

Critical illness induces severe muscle wasting at a loss of 2% per day in the first week of critical illness [1]. Muscle wasting is associated with poorer functional recovery and ultimately worse short- and long-term outcomes such as impaired immune function and resistance to infections, delayed wound healing, respiratory failure, and weakness [2, 3].

During critical illness, total body protein losses occur rapidly over the first ten days, with the greatest losses in the first 1–2 days, as observed in a trauma cohort [4]. Several observational cohort studies have reported associations between increased protein intake and reduced mortality in critical illness [5–7]; however, these data are likely to be influenced by survival bias. Additionally, whole-body physiological data demonstrates an increase in protein synthesis with greater doses of dietary protein [8–10].

Accordingly, ICU guidelines (based on low quality evidence) recommended higher protein doses than those recommended in health, aiming for daily protein targets of 1.3 g/kilogram body weight/day delivered progressively [11] or 1.2–2.0 g/kg/day [12]. Yet in practice, average protein delivery may be as low as half of these recommended amounts [13, 14]. Trials that aim to define the amount of protein to deliver during critical illness to optimise outcomes were therefore warranted. This manuscript aims to provide clinicians with advice to interpret the available evidence into clinical practice recommendations for use at the bedside. This advice is based on more contemporary evidence than has been incorporated into the most recent international ICU nutrition guidelines and are likely to evolve as more evidence is available.

## Main text

### Determining protein dose

In healthy cohorts, protein intakes of ~ 0.8 g/kg/day have been recommended [15, 16]. Rates of protein synthesis and catabolism are usually in balance, with various processes ensuring body protein stores are ‘spared’ if protein intake is low [17]. These recommendations have been based on nitrogen balance studies in young adults receiving dietary protein of high biological value and digestibility, with individual requirements affected by many factors including variation in growth, activity, environment and diet quality [17].

In critically ill patients, determining protein needs is complicated by comorbidities as well as dynamic physiological and metabolic changes that transpire following illness or injury [18]. Increased protein synthesis is outweighed by a simultaneous increase in catabolism leading to a negative protein balance on both the whole body and muscle level [19]. Furthermore, the protection of body protein stores is reduced by significant metabolic alterations, insulin resistance, and decreased physical activity [20–22]. Individual requirements may vary widely, potentially influenced by different clinical diagnoses, comorbidities, comedications, and inflammatory response [23].

### Summary of large trials to date

Recently, three high quality large-scale randomised controlled trials (RCT) of protein dose in critically ill patients have been conducted, each asking a slightly different question about protein dose [24–26] (Table 1). Overall, these three trials evaluated 5633 patients with median delivered protein doses per trial ranging between 0.69 and 1.19 g/kg/day in the lower protein group and 1.12–1.87 g/kg/day in the higher protein group.

The first of these trials, EFFORT Protein, was a parallel group pragmatic registry-based RCT which compared higher dose protein prescription (≥ 2.2 g/kg/day) to usual dose protein prescription (≤ 1.2 g/kg/day) in 1301 invasively mechanically ventilated patients at nutritional risk, defined as the presence of: body mass index (BMI) ≤ 25 or ≥ 35 kg/m^2^, moderate-severe malnutrition, frailty, sarcopenia risk, or projected duration of mechanical ventilation of more than 4 days [25]. Protein targets were met using any combination of enteral or parenteral nutrition, intravenous amino acids, or enteral protein supplements as per local standards of care. Protein doses achieved were median [IQR] 1.7 [1.3-2.0] versus 1.0 [0.8–1.1] g/kg/day. The number of nutrition evaluable days post-randomisation censored at day 12 was 11 [6–11] versus 10 [6–11] days. There was no statistically significant difference in the primary outcome of cumulative incidence of alive hospital discharge to day 60 between groups (46.1% vs. 50.2%; HR 0.91; 95% CI 0.77–1.07; *P* = 0.27). Bayesian analyses under weakly-informative priors suggested that higher protein had a 92% probability of harm with respect to time-to-discharge alive from the hospital and a 72% probability of harm with respect to 60 day mortality [27]. Of the 11 subgroup analyses, higher protein was associated with worse outcome in those patients with an acute kidney injury (AKI), as defined by Kidney Disease: Improving Global Outcomes (KDIGO) criteria, and high SOFA score (≥ 9) at baseline.

Published in 2024, PRECISe was a quadruple-blinded parallel group RCT which compared protein targets of 2.0 g/kg/day to 1.3 g/kg/day, delivered through isocaloric enteral formula in 935 invasively mechanically ventilated patients. Patients received median protein doses of 1·87 [0·96 − 2·00] versus 1·19 [0·63 − 1·26] g/kg/day, delivered over a median [IQR] of 10 [5–21] versus 9 [4–19] days. For the primary outcome, higher protein dose led to worse health-related quality of life to day 180, as quantified using the EQ-5D-5L health utility score, where death was assigned a score of 0 (mean diff − 0·05; 95% CI -0·10, -0·01; *P* = 0·031) [24]. Time-to-discharge alive was also longer in patients in the higher protein group (0·91 (95% CI 0·80, 1·04; *P* = 0.043). Of the 13 subgroups analysed, higher protein was associated with worse outcome in female patients and those admitted for medical diagnoses (compared to surgical admissions).

Most recently, TARGET Protein, a cluster-randomised, double crossover, open label clinical trial compared isocaloric enteral nutrition formulae with augmented protein (100 g protein/L) to a high protein formula (63 g protein/L) in 3397 ICU patients. Median protein doses achieved were 1.12 [0.71–1.53] vs. 0.69 [0.40–0.95] g/kg ideal body weight/day (including all feeding days), delivered for a median of 87 [36–187] versus 84 [34–182] hours. For the primary outcome, there was no statistically significant difference in the number of days free of the index hospital and alive at day 90 between groups (62 (0–77) vs. 64 (0–77) days, adjusted-for-period between-group median diff − 1.97 (95% CI − 7.24 to 3.30) days; *P* = 0.46) [26]. Of the four subgroups analysed, there was heterogeneity of treatment effect in patients with invasive mechanical ventilation and new kidney replacement therapy favouring usual protein in both groups.

In addition, eight systematic reviews with meta-analyses of RCTs that investigated the effect of higher protein delivery in the critically ill have been published in the last ten years [28–35]; four of these compared protein doses within international recommendations (≥ 1.2 g/kg/day) to below recommendations (< 1.2 g/kg/day) [29, 32, 33, 35]. These eight reviews collectively indicate that higher protein delivery during critical illness has not demonstrated benefit on mortality or duration of ICU and hospital admission. In particular, the systematic review and meta-analysis by Heuts et al., incorporating both EFFORT Protein and PRECISe, used a Bayesian analysis approach to report that the probability of any mortality benefit from higher protein delivery was 43.6%, while the probability of harm was 56.4% [34]. While more recently, Summers et al. incorporated all three large RCTs and concluded that greater protein delivery did not affect mortality (pooled RR 1.01, 95% CI 0.92, 1.12, *P* = 0.795; I^2^ = 0%; τ^2^ = 0.00; 12 RCTs: greater protein *n* = 3197; lesser protein *n* = 3243) [35].

In summary, no large trial has shown benefit of augmented protein over a standard protein approach in critically ill patients across a range of available doses. In interpreting this evidence, it is important that clinicians recognise that while protein targets were set at up to ≥ 2.2 g/kg/day, protein doses achieved were much less, reflective of routine clinical care where protein delivery does not equate to protein prescriptions [13, 36]. Further to this, clinicians should recognise that heterogeneity does exist between trials as highlighted in Table 1. Differences in these trials include the methodological approaches used, patient populations studied, and outcomes evaluated. Importantly, despite this heterogeneity between trials, the findings for lack of benefit for higher protein were consistent, which increases the generalisability of results.

### Specific populations to consider

#### (Acute) Kidney Injury

In patients with (acute) kidney injury, data from five RCTs suggest that the provision of higher protein doses is not beneficial and might be harmful [24–26, 37–39]. Subgroup data from the recent RCTs provide inconsistent results (Table 2). EFFORT Protein found that higher protein delivery was associated with worse outcomes (discharge-alive-from-hospital at day 60 (HR 0.5, 95% CI 0.4, 0.8) and 60-d mortality (RR 1.4, 95% CI 1.1, 1.8)) compared to lower doses, in patients who developed AKI (*n* = 312) [25], while these findings were not replicated in PRECISe (*n* = 188) [24]. While both trials used the KDIGO criteria to define an AKI, EFFORT Protein included those meeting these criteria within 7 days of ICU stay (prior to randomisation occurring up to 96 h after ICU admission), whereas PRECISe identified an AKI at time of randomisation only (within 24 h of ICU admission). Of note, a recent systematic review and meta-analysis concluded with high certainty that higher protein is associated with increased mortality in patients with an AKI (RR 1.42, 95% CI 1.11, 1.82), noting that different definitions for AKI or timepoints at which they were assessed were used in each trial [31].

In patients receiving new kidney replacement therapy in the ICU prior to commencement of trial enteral nutrition (*n* = 241), TARGET Protein found moderate credibility that higher protein was associated with harm for days free of the index hospital and alive at day 90 [26]. Conversely, in a post-hoc analysis of EFFORT Protein, these suggested harmful effects of higher protein dose in AKI did not persist when patients received kidney replacement therapy [40].

Overall, the heterogeneous definition of AKI and timing of kidney replacement therapy in various trials complicates the interpretation of the results. These data suggest that recommendations for higher protein supplementation in AKI patients may need to be re-evaluated to avoid potential harm, particularly in those who are not receiving kidney replacement therapy. As such, we suggest providing a maximum of 1.2 g protein/kg/day to patients with an AKI at any stage of illness. Given the presence of a potential signal for harm in this cohort in some studies, we suggest that these patients be monitored with greater caution.

#### Obesity

Protein dosing for critically ill patients with obesity may require special consideration due to altered body composition and metabolic demands. The ASPEN 2016 guidelines recommended greater protein doses for patients with obesity of up to 2.5 g/kg ideal body weight/day [41], informed by one small RCT in hospitalised patients with obesity (*n* = 30) conducted nearly 30 years ago [42] and observational data [43]. In PRECISe, there was no interaction between BMI < 30 vs. ≥ 30 kg/m^2^ and the response to higher protein targets with respect to health-related quality of life or overall survival [24]. This is also supported by the lack of benefit in the TARGET Protein subgroup analysis of BMI < 35 vs. ≥ 35 kg/m^2^ [26]. Similarly, a post-hoc analysis of EFFORT Protein, that recruited patients at nutritional risk, found no significant benefit of high protein dose in a subgroup of those with obesity (BMI < 30 vs. ≥ 30 kg/m^2^) [44]. Together, these results provide a signal that patients with obesity are no more likely to benefit from higher protein doses than patients without obesity in critical illness, contrary to what is proposed in the most recent international guidelines available for this patient cohort. It remains unclear whether protein dosing for patients with obesity (or any patient for that matter) should be based on an estimation of skeletal muscle mass rather than total or ideal body weight to avoid unnecessary protein overfeeding.

#### Other populations of interest

International guidelines also recommend higher protein doses for patients with clinical conditions where it is assumed higher protein intake is needed, but with limited evidence. The ASPEN 2016 guidelines [41] and ESPEN 2013 guidelines [45] advocate 1.5–2.0 g protein/kg/day or even higher in patients with severe burns to counteract hypercatabolism and to meet protein losses, despite low quality evidence for high protein diets in this cohort [46]. Similarly, ASPEN guidelines advocate for early (24–48 h) high protein polymeric enteral nutrition in stable patients following major trauma within 1.2–2.0 g/kg/day [41], yet a recent RCT in 500 critically ill trauma and surgical patients indicated that high enteral protein supplementation did not improve outcomes but was associated with increased incidence of reintubation [47]. Therefore, until more robust evidence is available, standard protein targets (approximately 1.3 g/kg/day) may be considered, and we suggest close monitoring of wound healing and adaptation of protein dose accordingly.

### Reasons for lack of benefit and presence of harm with higher protein dose

The emerging evidence for the lack of benefit and presence of harm with higher protein delivery in critical illness logically drives the quest for possible explanations. Both from a biological point of view as well as derived from prospective mechanistic and exploratory post-hoc studies, there are some potential explanatory mechanisms. It also serves as an important reminder to continually seek the highest quality evidence within all areas of clinical practice.

**Fig. 1:**
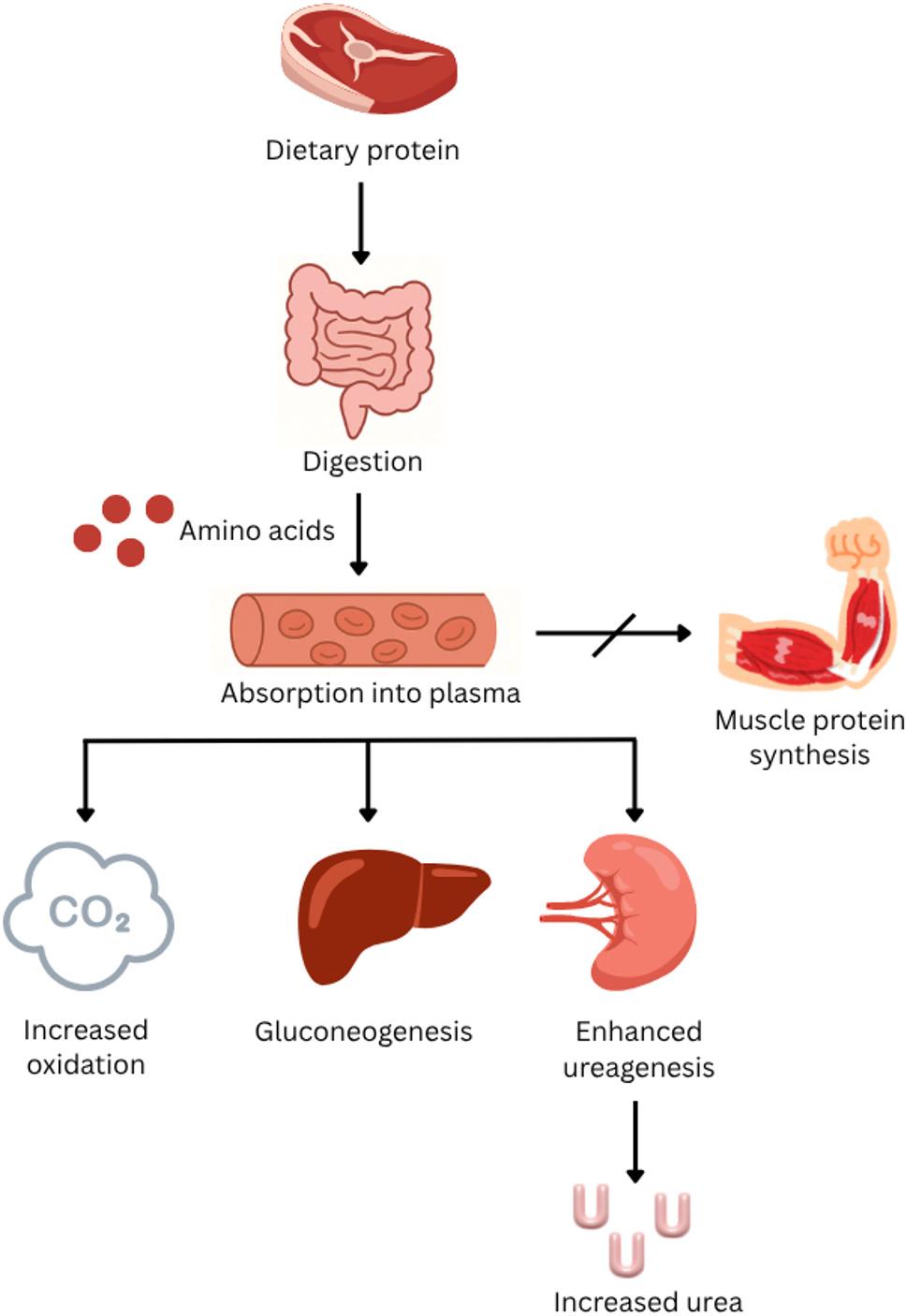
Processes of amino acid disposal in health and critical illness

Using stable isotopes, a landmark study demonstrated that critically ill patients exhibit impaired rates of post-prandial muscle protein synthesis compared to healthy volunteers [48]. This phenomenon, called anabolic resistance to dietary protein, poses a great challenge in that it not only impairs muscle synthesis, but conversely leads to an increase in the intravenous free amino acid pool. Further work from this group on the dose-response relationship between enteral protein administration and muscle protein synthesis has recently been published [49]. This study demonstrated that higher doses of dietary protein are not able to further augment postprandial muscle protein synthesis rates to overcome anabolic resistance, despite increased amino acid availability in the bloodstream. When protein intake surpasses the physiological need, the excess amino acids are disposed of by three major processes: increased oxidation (production of carbon dioxide and ammonia), enhanced ureagenesis (production of urea), and/or gluconeogenesis [50] (Fig. 1).

While there is thought that non-protein energy sufficiency may be required to maximise the anabolic potential of amino acids [51], similar energy doses achieved between the protein groups in each individual trial provide reassurance that observed results are due to different protein doses and not energy delivery (Table 2).

An increased urea load has recently been demonstrated in all three large protein dose trials [24–26]. A post-hoc analysis of EFFORT Protein more closely analysed the relationship between the high protein intervention, serum urea trajectories, and 30-day mortality using combined Cox and linear mixed models [27]. Adjusted for confounding factors, it was calculated that higher urea over time was directly associated with higher mortality (HR 1.34; 95% CI 1.21, 1.48), as opposed to a non-significant effect for the protein intervention itself. Higher doses of protein thus resulted in higher urea trajectories which were in turn associated with an increased mortality risk. This indication requires testing in prospective work.

In addition, urea can be related to serum creatinine, leading to the urea-to-creatinine ratio (UCR). This ratio is increasingly highlighted as a potential marker for protein catabolism and indicator of muscle wasting, since creatinine decreases as muscle protein is broken down [52]. A recent scoping review found that an increased UCR occurred in patients with a longer length of ICU, as well as in patients with greater muscle loss [53]. One guideline recommended calculating UCR routinely, especially in patients with pre-existing malnutrition, sarcopenia, or an expected treatment duration of ≥ 7 days [54]. However, UCR does not fully reflect muscle protein metabolism, can be difficult to interpret in certain clinical scenarios (e.g. gastrointestinal bleeding, patients receiving kidney replacement therapy or diuretics), and there is no evidence that this translates to improved outcomes. Future prospective data are needed, and additional work should explore how UCR could be implemented in routine practice to guide protein dosing in individual patients.

Specific to the AKI cohort, reasons for potential harm may relate to excessive early protein administration overwhelming metabolic capacity, leading to azotemia, hyperglycemia, and increased hepatic/renal burden [55]. As mentioned above, additional amino acids unable to be utilised for muscle protein synthesis [32] may be converted to urea, increasing the serum urea level [22, 23, 24]. The increased urea concentration cannot be effectively cleared by patients with AKI who are not dialysed, increasing the metabolic burden and leading to increased mortality risk [31, 39]. This may explain the signal for harm disappearing in some trials when kidney replacement therapy is commenced [40], due to a reduction in renal burden.

### What future data might tell us

Data to date demonstrate that higher protein doses delivered in critical illness have not provided benefit for clinical or functional outcomes. The actively recruiting REPLENISH trial will contribute further data on protein dose in critical illness. REPLENISH is a multi-centre RCT in 2500 patients that uses a protocolised step-wise approach to achieve higher protein dose (2.0–2.4 g/kg/day) commenced only from day 5 in ICU compared to usual protein dose (0.8–1.2 g/kg/day) on the primary outcome of all-cause 90-day mortality [56]. While REPLENISH will inform about high protein doses compared to usual protein delivery, additional research should also determine whether even lower doses of protein than those received are safe or even beneficial.

While patients with a longer ICU length of stay were included in all three trials (EFFORT Protein, PRECISe and TARGET Protein), the median duration of protein delivery was no more than 11 days. As physiological data demonstrate improved muscle protein synthesis over time in long-stay ICU patients [57], further data is required to identify if there is a point in the illness trajectory at which higher protein doses provide benefit to functional recovery before protein doses are increased in practice. It seems conceivable that higher protein doses could become beneficial when a patient is capable of active mobilisation or physical exercise which may result in an anabolic stimulus. Furthermore, it remains unknown whether higher protein doses provided to patients after ICU discharge elicits benefit.

In addition, patient cohorts that are likely to benefit, or be more vulnerable to higher protein doses, are important to elicit as we move toward understanding individualised treatment effects [58]. Some considerations include the impact of baseline nutritional status on responsiveness to protein [59], or inflammatory phenotypes that might influence protein utilisation [59, 60].

### What should I do in practice?

**Fig. 2:**
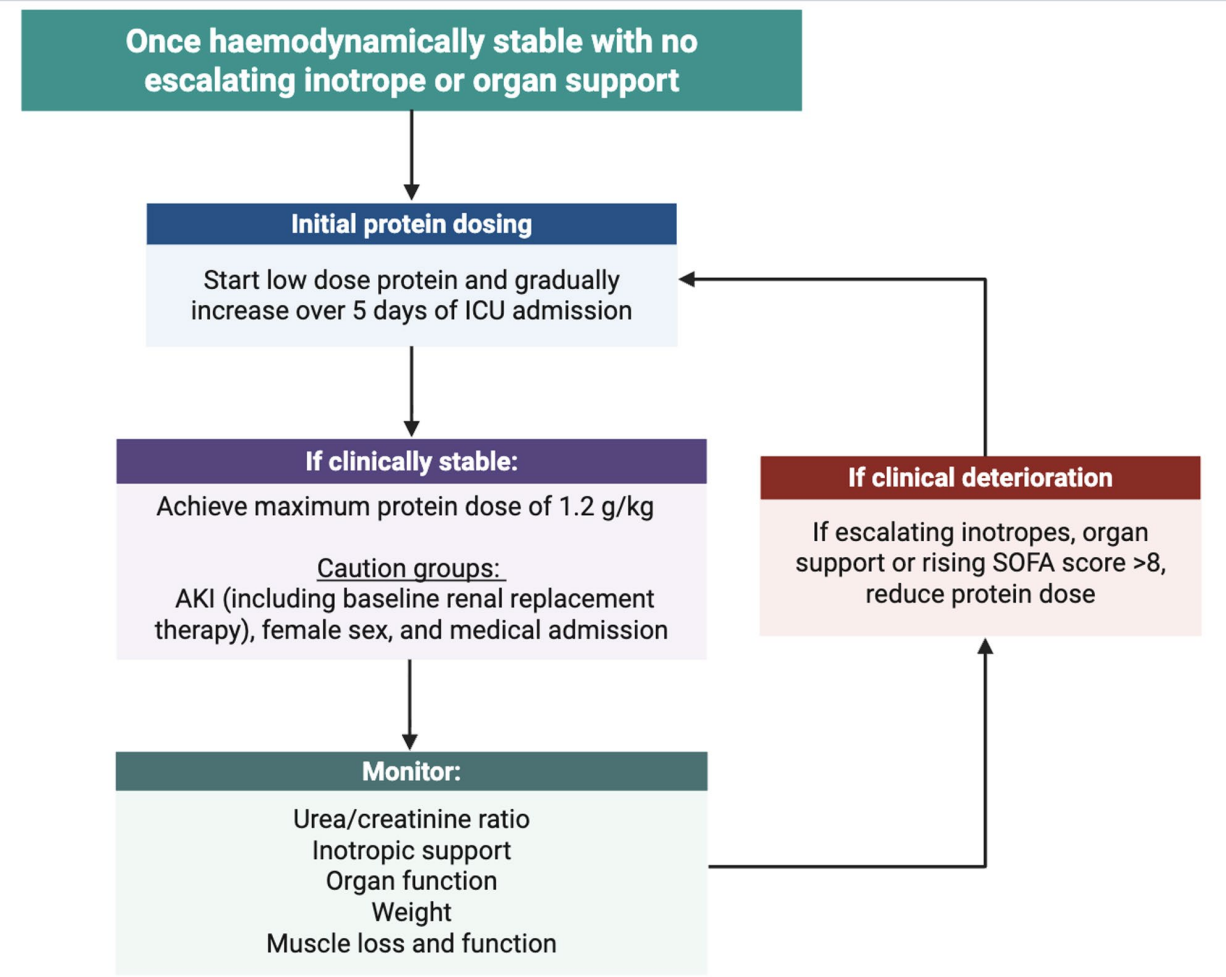
How protein dose trials may be incorporated into clinical practice

Bringing all the presented evidence into practice is a clinical challenge. Table 3 provides suggestions for protein delivery in critical illness and the level of evidence that supports each suggestion, and Fig. 2 provides a visual summary of how clinicians might practically implement the advice provided within this paper. We suggest that nutrition be gradually introduced over 2–3 days and only progressed when a patient is considered hemodynamically stable with no escalating inotrope or organ support to targets, by around day 5. Once progression is possible, the maximum dose of protein to be delivered to the patient that we suggest is 1.2 g/kg/day in ICU. It must be recognised that only approximately 60% of what is prescribed, is actually delivered to the patient [36], but we do not advocate for the use of higher protein content formulae to achieve these protein doses. Regular monitoring of clinical status (organ function, inotrope support), laboratory parameters, feeding tolerance, and weight and muscle loss/function may help to inform protein dose. Sub-populations where harm has been suggested should likely be carefully considered (i.e. those with AKI, female sex, and medical admission categories), as the maximum dose tolerated may be even less than for other patient cohorts. Conversely, there is no current evidence to treat other sub-populations differently in the acute phase of illness and any reasons to do so should be cautiously reviewed. Limited evidence exists to inform protein dosing for patients with a very prolonged ICU admission, or in the post-ICU period; the timeframe for recovery is unclear.

## Conclusions

Recent large trials demonstrate that higher protein doses do not benefit clinical outcomes such as those incorporating survival and length of stay, and may worsen recovery and quality of life. Protein doses may affect patient subgroups differently: patients with an AKI may be particularly vulnerable. In the absence of further evidence, we suggest that for now clinicians aim to limit protein delivery to a maximum of 1.2 g protein/kg/day during the ICU stay, recognising this as a pragmatic upper boundary based on the currently available evidence until further evidence defines optimal intake. Future research is needed to determine the safest protein dose to provide during critical illness, and the optimal protein dose to provide after ICU discharge to improve patient recovery.

Take home message:

- Progressive delivery of protein doses to a maximum of 1.2 g/kg/day appears to be safer than higher doses for most patients. The optimal dose is unknown, and may be less than 1.2 g/kg/day.
- Additional caution should likely be applied to patients with an acute kidney injury.
- Subgroup analyses have not consistently shown benefit of higher protein doses in specific patient populations.

**Table 1:** Comparison between definitive randomised controlled trials of protein dose in critically ill patients

	EFFORT Protein [25]	PRECISe [24]	TARGET Protein [26]
	**METHODS**		
**Study design**	Single-blind parallel group RCT	Quadruple-blinded, parallel group RCT	Cluster randomised, double cross-over, pragmatic, open-label RCT
**Sites**	85 ICUs from 16 countries	10 ICUs from Netherlands and Belgium	8 ICUs from Australia and New Zealand
**Population** **(key inclusion)**	-≥18 years -IMV -Nutritionally high risk	-≥18 years -Unplanned ICU adm -IMV initiated < 24 h following ICU adm -Expected ICU stay on ventilator support for ≥3d	*-*≥16 years of age -Receiving/about to commence EN during their index admission to ICU or receiving/about to commence EN for the first time in ICU during subsequent admissions
**Intervention group**	High dose protein ≥ 2.2 g/kg/day*	High dose enteral protein (8 g protein/100 kcal) delivered to achieve 2.0 g/kg/day^#^	Provision of EN formula (100 g protein/L)
**Comparator group**	Usual dose protein ≤ 1.2 g/kg/day*	Standard dose enteral protein (5 g protein/100 kcal) delivered to achieve 1.26 g/kg/day^#^	Provision of EN formula (63 g protein/L)
**Duration of feeding**	Within 96 h of ICU adm until a maximum of 28d, or discharged from ICU, or dies	Within 48 h of ICU adm, continued for the duration of ICU stay if EN is required or until a maximum of 90d	Administered when clinically indicated in ICU (within 12 h of commencing EN) until a maximum of 90d, or discharged from ICU, or dies
**Primary outcome**	Time-to-discharge-alive from hospital up to 60d after ICU adm	EQ-5D-5L health utility score over three time-points (30, 90, and 180d after ICU admission), adjusted for baseline EQ-5D-5L	Number of days free of the index hospital and alive at day 90
	**RESULTS**		
**Patient cohort**	1301 patients (645 intervention vs. 656 comparator)	935 patients (470 intervention vs. 465 comparator)	3397 patients (1681 intervention vs. 1716 comparator)
**Protein dose delivered**	Median (IQR): 1.7 (1.3-2.0) vs. 1.0 (0.8–1.1) g/kg/d	Median (IQR): 1·87 (0·96 − 2·00) vs. 1·19 (0·63 − 1·26) g/kg/d	Median (IQR): All feeding days: 1.12 (0.71–1.53) vs. 0.69 (0.40–0.95) g/kg IBW/d Excluding day of enrolment: 1.33 (0.88–1.78) vs. 0.84 (0.49–1.11) g/kg IBW/d
**Energy dose delivered**	Mean (SD): 14·7 (6.9) vs. 13·2 (6·4) kcal/kg/day	Median (IQR): 24 (12–25) vs. 24 (13–25) kcal/kg/day	Median (IQR): 14 (9–19) vs. 14 (8–19) kcal/kg IBW/day
**Duration of feeding**	Intervention vs. comparator: 11.0 [6.0–11.0] vs. 10.0 [6.0–11.0] d Stated as number of nutrition evaluable days post-randomisation (maximum 12 days)	Intervention vs. comparator: 10 [5–21] vs. 9 [4–19] d	Intervention vs. comparator: 87 [36–187] vs. 84 [34–182] h
**Primary outcome result**	No difference in cumulative incidence of alive hospital discharge to day 60 between group: 46.1% vs. 50.2% (HR 0.91; 95% CI 0.77–1.07; *P* = 0.27)	Worse EQ-5D-5L health utility score with intervention (mean diff − 0·05; 95% CI -0·10 to -0·01; *P* = 0·031)	No difference in median [IQR] number of days free of the index hospital and alive at day 90 between intervention and comparator: 62 (0–77) vs. 64 (0–77) d, adjusted-for-period between-group median diff − 1.97 (95% CI − 7.24 to 3.30) d; *P* = 0.46
**Key secondary results**	No between-group differences in: -Cumulative incidence of alive hospital discharge to day 60 -60d mortality -Hospital mortality -Duration of IMV -ICU length of stay -Hospital length of stay	No between-group differences (to day 180): -SF-36 -Hospital anxiety and depression scale -Impact of event scale-revised -MRC-Sum score -EQ-5D-5L VAS -Rockwood clinical frailty scale Intervention associated with: -Higher incidence of GI intolerance -Greater use of prokinetic drugs -Longer duration of stay in a rehabilitation facility -Improved % predicted 6 min walk test -Longer time to discharge alive (post-hoc)	No between-group differences in: -Survival at day 90 -Median days free of hospital in survivors -Mean duration of IMV -Time to live hospital discharge -Tracheostomy insertion -New kidney replacement therapy -Discharge destinations Longer time to live ICU discharge in favour of usual protein
**Subgroup analyses**	11 subgroups. Intervention associated with worse outcome in patients with AKI and high SOFA score (≥ 9) at baseline	13 subgroups. Intervention associated with worse outcome in female patients and medical admissions	4 subgroups. Intervention associated with worse outcome in patients with IMV and new kidney replacement therapy (heterogeneity of treatment effect)
**Trial strengths**	Geographically diverse sites Heterogenous population Population enrichment (‘high’ nutritional risk)	Blinded Controlled protein intervention Functional recovery primary outcome	Composite outcome (hospital length of stay, mortality and readmission) Large cohort Controlled protein intervention
**Trial limitations that impact interpretation**	Protein route and source not controlled Calorie intake not controlled (but similar between groups)	9.2% loss to follow-up for the primary outcome, and higher for secondary outcomes	Open label Minimal data collected on protein intake Short term nutrition delivery (median < 4 days)

*Set using pre-ICU actual dry weight, or for patients with a BMI >30 kg/m², an ideal bodyweight based on a BMI of 25 kg/m² was used

^#^Set using adjusted body weight with BMI>27 (weight calculated back to BMI=27 for length)

adm=admission; AKI=acute kidney injury; CI=Confidence interval; d=day; EN=Enteral nutrition; EQ-5D-5L=EuroQol-5 dimensions-5 levels; h=hours; IBW=Ideal body weight; ICU=Intensive care unit; IMV=Invasive mechanical ventilation; RCT=Randomised controlled trial; SOFA=Sequential organ failure assessment; und=undefined; VAS=Visual analogue score

**Table 2:** Clinical outcomes of patients with acute kidney injury

RCTs	*n*	Definition of “Acute Kidney Injury”	*n* with AKI	Outcome (higher vs. lower protein)
Singer 2007 [38]	14	50% decrease in GFR, a doubling of serum creatinine or an increase of creatinine to 3.5 mg/dL (309.4 umol/L).	14	ICU mortality: 3/8 (37.5%) vs. 2/6 (33.3%)
Doig 2015 [37] Zhu 2018 [39]	471	• **Baseline Kidney Dysfunction**: Creatinine at time of enrolment > 168µmol/L (by Gordon Bernard’s *“*Brussels Table”), or • **Risk of progression of AKI at enrollment**: rise in creatinine over the previous 24 h by at least 20% to over 120 µmol/L	106	90-d mortality: 17/60 (28.3%) vs. 7/46 (15.2%) - Interaction relative risk: 3.57 (1.16–11.0)
Heyland 2023 [25]	1290	Patients who met the KDIGO criteria: • Stage 1: at least 26·52 µmol/L increase in serum creatinine from baseline within 48 h or 1·5–1·9 times baseline within 7d • Stage 2: 2·0–2·9 times baseline within 7d • Stage 3: 3 times or more baseline within 7d or increase to at least 353·6 µmol/L with an acute increase of more than 44·2 µmol/L.	312	Time-to-discharge-alive from hospital up to day 60: - Hazard ratio: 0.5 (0.4– 0.8) 60-d mortality: 82/162 (50.6%) vs. 53/146 (36.3%) - Relative risk: 1.4 (1.1–1.8)
Bels 2024 [24]	935	KDIGO score of 1 or more, calculated at randomisation	199	Mortality up to day 180: - Hazard ratio 1.05 (0.72– 1.53)
Summers and Chapple 2025 [26]	3397	Receiving new kidney replacement therapy between hospital admission and commencement of trial enteral formula on ICU admission	241	No. of days free of the index hospital and alive at day 90: - Median difference: -13.19 (-50– 23.62)

AKI=acute kidney injury; d=day; GFR= Glomerular Filtration Rate; ICU= intensive care unit; KDIGO= Kidney Disease: Improving Global Outcomes

**Table 3:** Clinical recommendations and supporting evidence for protein dosing in critically ill adults

Clinical recommendation	Supporting evidence for recommendation:
We suggest commencing protein delivery at a low dose from ICU admission and increasing progressively over the first 5 days of admission to provide **a** ***maximum*** **of 1.2 g/kg/day to** critically ill adult patients over the ICU admission. The use of higher protein content formulae or increasing protein-to-energy ratio to achieve these targets are not recommended.	*Primary outcomes*: - PRECISe: Worse quality of life to day 180 in patients receiving 1.87 versus 1.19 g protein/kg/day [24]
	- TARGET Protein: No difference in the number of days free of the index hospital and alive at day 90 in patients receiving 1.12 versus 0.69 g protein/kg/day [26]
	*Secondary outcomes*:
	- TARGET Protein: Longer time to live ICU discharge in patients receiving 1.12 versus 0.69 g protein/kg/day [26]
	*Secondary analyses*:
	- EFFORT Protein: Bayesian analyses under weakly-informative priors showed 92% probability of harm with respect to time-to-discharge alive from the hospital with 1.7 versus 1.0 g protein/kg/day [61]
	- EFFORT Protein: Bayesian analyses under weakly-informative priors showed 72% probability of harm with respect to 60 day mortality with 1.7 versus 1.0 g protein/kg/day [25]
	- PRECISe: Bayesian analyses showed posterior probability of benefit from high (2.0 g/kg/day) protein targets with respect to the EQ-5D-5L health utility score was 0% [62]
	*Meta-analysis*:
	- 56.4% probability of harm for mortality from higher protein delivery [34]
Evidence is inconclusive, but in patients with an acute kidney injury, we suggest limiting protein doses to a maximum of 1.2 g/kg/day with close monitoring.	*Subgroup analyses*: - EFFORT Protein: Longer discharge-alive-from-hospital at day 60 and higher 60 day mortality in those patients with an acute kidney injury (AKI) as defined by Kidney Disease: Improving Global Outcomes (KDIGO) criteria receiving 1.7 versus 1.0 g protein/kg/day [25]
	*Meta-analysis*:- High certainty of increased mortality with higher protein in patients with an AKI [31]
Evidence is inconclusive, but in patients with an acute kidney injury requiring kidney replacement therapy, we suggest limiting protein doses to no more than 1.2 g/kg/day.	*Subgroup analysis*: - TARGET Protein: Moderate credibility that higher protein (1.12 versus 0.69 g protein/kg/day) is associated with harm for days free of the index hospital and alive at day 90 in patients receiving new kidney replacement therapy from ICU admission to *prior to randomisation *[26]
	*Post-hoc analysis*:
	- EFFORT Protein: Harmful effects of higher protein dose in AKI did not persist when patients received kidney replacement therapy after randomisation [40]
Evidence is inconclusive, but it is probable that patients with obesity are no more likely to benefit from higher protein doses than patients without obesity in critical illness, such that doses of no more than 1.2 g/kg/day are suggested for this cohort	*Subgroup analyses*: - PRECISe: No interaction between BMI < 30 vs. ≥ 30 kg/m^2^ and the response to higher protein targets with respect to health-related quality of life or overall survival [24]
	- TARGET Protein: No difference in number of days free of the index hospital and alive at day 90 with 1.12 versus 0.69 g protein/kg/day in patients BMI < 35 vs. ≥ 35 kg/m^2^ [26]
	*Post-hoc analysis*:
	- EFFORT Protein: No significant benefit of high protein dose in a subgroup of those with obesity (BMI < 30 vs. ≥ 30 kg/m^2^) [44]
Evidence is inconclusive, but protein targets to a maximum of 1.2 g/kg/day may be considered for patients with major trauma until definitive evidence available	No definitive evidence to support higher protein dose recommendations (1.5–2.0 g protein/kg/day) in international critical care nutrition guidelines for patients with major trauma [47]
Evidence is inconclusive, but subgroups that may require lower protein doses that may warrant monitoring with caution include: female sex, medical inpatients, higher SOFA at baseline	*Subgroup analyses*: - PRECISe: Worse quality of life to day 180 with higher protein in female compared to male patients [24]
	- PRECISe: Worse quality of life to day 180 with higher protein in those admitted for medical diagnoses (compared to surgical admissions) [24]
	- EFFORT Protein: Longer time-to-discharge-alive and higher 60-day mortality with higher protein in patients with a higher SOFA score (≥ 9 versus < 9) on admission [25]

Recommendations informed by lower quality evidence, including secondary, subgroup, or post-hoc analyses, should be taken as suggestions only, and require prospective validation

## Data Availability

No datasets were generated or analysed during the current study.
